# Oligonucleotide Formulations Prepared by High-Speed Electrospinning: Maximizing Loading and Exploring Downstream Processability

**DOI:** 10.3390/pharmaceutics15030855

**Published:** 2023-03-06

**Authors:** Edit Hirsch, Márió Nacsa, Eszter Pantea, Edina Szabó, Panna Vass, Júlia Domján, Attila Farkas, Zoltán Nyíri, Zsuzsanna Eke, Tamás Vigh, Sune Klint Andersen, Geert Verreck, György János Marosi, Zsombor Kristóf Nagy

**Affiliations:** 1 Department of Organic Chemistry and Technology, Budapest University of Technology and Economics, Müegyetem rkp. 3, 1111 Budapest, Hungary; 2 Joint Research and Training Laboratory on Separation Techniques, Eötvös Loránd University, Pázmány Péter stny. 1/A, 1117 Budapest, Hungary; 3 Oral Solids Development, Janssen R&D, Turnhoutseweg 30, B-2340 Beerse, Belgium

**Keywords:** biopharmaceutical formulation, electrospinning, solvent optimization, downstream processing, high-drug-loaded fibers, antisense oligonucleotide, oral delivery

## Abstract

The aim of this study was to develop antisense oligonucleotide tablet formulations using high-speed electrospinning. Hydroxypropyl-beta-cyclodextrin (HPβCD) was used as a stabilizer and as an electrospinning matrix. In order to optimize the morphology of the fibers, electrospinning of various formulations was carried out using water, methanol/water (1:1), and methanol as solvents. The results showed that using methanol could be advantageous due to the lower viscosity threshold for fiber formation enabling higher potential drug loadings by using less excipient. To increase the productivity of electrospinning, high-speed electrospinning technology was utilized and HPβCD fibers containing 9.1% antisense oligonucleotide were prepared at a rate of ~330 g/h. Furthermore, to increase the drug content of the fibers, a formulation with a 50% drug loading was developed. The fibers had excellent grindability but poor flowability. The ground fibrous powder was mixed with excipients to improve its flowability, which enabled the automatic tableting of the mixture by direct compression. The fibrous HPβCD–antisense oligonucleotide formulations showed no sign of physical or chemical degradation over the 1-year stability study, which also shows the suitability of the HPβCD matrix for the formulation of biopharmaceuticals. The obtained results demonstrate possible solutions for the challenges of electrospinning such as scale-up and downstream processing of the fibers.

## 1. Introduction

Antisense oligonucleotides (ASOs) are short synthetic strings of nucleotides designed to alter the expression of a protein involved in human diseases by selectively binding to its mRNA. Since the first report of antisense-mediated gene suppression about 30 years ago, the interest in ASOs as pharmaceutical agents for the treatment of neurological and cardiovascular diseases, cancers, various infections, inflammatory, and metabolic diseases has increased [1,2,3].

Despite the therapeutic potential of ASO-based therapies, there are difficulties in delivery because they tend to break down by endonucleases in the bloodstream and within cells. Furthermore, fast renal clearance and inefficient cell entry can result in low efficiency. To overcome these limitations, second- and third-generation ASOs were developed that have improved target affinity, nuclease resistance, stability, and pharmacokinetic properties [4,5]. These can be achieved by the chemical modification of the RNA molecule and conjugation to cell/tissue-targeting ligands. In combination with these strategies, several drug delivery systems were developed including the use of penetration enhancers, lipid and polymeric nanoparticles, and liposome complexes [6,7,8,9,10].

The development of advanced oligonucleotide chemistry and innovative drug delivery strategies made it possible to target local delivery of ASOs using oral drug formulations [11,12,13,14,15]. Advantages include high local concentration of the active pharmaceutical ingredient (API), smaller doses, and better patient compliance besides the lower toxicity and weaker adverse immunogenicity. Despite the advantages, currently, there are no oral tablets available on the market. However, the number of research studies with a focus on delivering oligonucleotides orally is growing rapidly [16]. For example, an oral delivery system of an ASO against tumor necrosis factor-α was developed utilizing triple helical β-glucan–poly deoxy adenylic acid nanoparticles in combination with a chitosan-alginate hydrogel system [17]; or the oral delivery of an oligonucleotide conjugated to N-acetylgalactosamine for targeting of PCSK9 in the liver, using a sodium-caprate-based tablet, was accomplished [18].

The use of biopharmaceuticals for oral delivery requires a stable formulation of the ASOs in a solid form. The solid formulation strategy offers several potential advantages, such as improvement in long-term stability, easy shipping, and decreased risk for microbial growth. Usually, freeze-drying is applied for the solid formulation of oligonucleotides [19,20,21,22]. However, freeze-drying exposes the API to freezing stresses, which can cause degradation (e.g., aggregation). The drying results in a cake, which needs additional processing (e.g., milling) in order to obtain a powder suitable for tableting. In addition, freeze-drying is considered to be expensive as it is an energy- and time-consuming process. In addition, it is usually operated in batch mode, which, coupled with a long cycle time, can be a bottleneck of the development process [23]. Therefore, in the last decade, numerous studies have focused on the development of continuous freeze-drying methods [24].

As an alternative to freeze-drying, electrospinning was shown to be an effective approach for the production of stable solid forms of sensitive biologicals including oligonucleotides [25,26,27]. Electrospinning technology is an efficient, continuous drying technology that utilizes the electrostatic forces on a liquid feed to generate fibers in a micro- and nano-range. The high surface area of the fibers enables ultra-fast drying even at room temperature. The characteristics of the applied solvent (e.g., volatility) and other processing parameters (e.g., feeding rate) highly influence the drying rate and the fiber morphology [28].

One of the main challenges of electrospinning is achieving a productivity suitable for commercial applications; furthermore, the continuous collection and the downstream processing of the fibers need to be addressed to meet the industrial requirements [29,30,31,32]. Pharma-industry-compatible high-speed electrospinning (HSES) technology was developed recently to increase production capacities utilizing centrifugal forces [33]. In addition, HSES connected to a cyclone was demonstrated to be a feasible solution for the continuous collection of fibers [34]. The conversion of electrospun fibers into oral tablets, as a final dosage form, requires adequate downstream processability (e.g., grindability), but the manufacture of tablets is especially challenging due to the physical properties of the fibers, such as low bulk density and poor flowability [35].

The main goals of the current research were to utilize HSES for the continuous production of ASO-containing fibers as an alternative to freeze-drying and to prepare a tablet dosage form as a possible oral drug formulation. The continuous collection and the downstream processing (grinding, blending, tableting) of the fibers were also examined during the development of hydroxypropyl-beta-cyclodextrin-based (HPβCD) formulations. Cyclodextrins are water-soluble cyclic oligomers consisting of α−1,4 D-glucopyranose units, which have been successfully used to produce electrospun fibers as it is capable of forming polymer-like supramolecular structures via intramolecular interactions [34,36,37]. Generally, for fiber formation of HPβCD, high concentrations are required to generate strong and large associates in the solution (resulting in high solution viscosity). Thus, different solution compositions (concentration, solvent type) were tested to evaluate their effect on solution viscosity and also product morphology. The ASOs used in this study are stable not only in pure water but also in methanol; hence, the development of HPβCD electrospinning was carried out using methanol, methanol/water (1:1), and water as solvents. Another main driver for the solid formulation development was to maintain the studied ASO’s long-term stability. Thus, the formulation’s stability was investigated during one year of storage at 4 °C and ~34% relative humidity. One of the greatest limitations of these compounds is that they have low oral bioavailability so that only a small amount of the ASOs reaches the target tissues unless the ASOs are used at very high doses. Thus, another aim of the study was to develop a high-loading formulation containing 50% API.

## 2. Materials and Methods

### 2.1. Materials

2-Hydroxypropyl-beta-cyclodextrin (HPβCD) (Kleptose^®^ HPB, MS nominal value: 0.62) purchased from Roquette Pharma (Lestrem, France) was used for electrospinning. Purified water prepared by reverse osmosis with a Zeneer UP S-UV (Human Corporation, Seoul, Republic of Korea) water system and analytical-grade methanol purchased from Merck. (Darmstadt, Germany) was used to prepare the solutions for electrospinning. Antisense oligonucleotides (ASO1 and ASO2) were kindly supplied by Janssen Pharmaceuticals.

For the tableting of ASO1-containing electrospun formulations microcrystalline cellulose (MCC, Vivapur 200) purchased from JRS Pharma (Rosenberg, Germany), mannitol (Pearlitol 400DC) kindly supplied by Roquette Pharma (Lestrem, France), croscarmellose sodium (Ac-Di-Sol) obtained from DuPont (Budapest, Hungary), silicon dioxide (Aerosil^®^ 200, Evonik Industries, Essen, Germany), and magnesium stearate provided by Hungaropharma (Budapest, Hungary) were used.

### 2.2. Viscosity Measurement

To measure the viscosity of the solutions used for electrospinning, an AR 2000 rotational rheometer (TA Instruments, New Castle, WY, USA) was used. The measurements were carried out in parallel plate configuration using a lower Peltier plate and a 40 mm stainless-steel upper moving plate. The temperature of the solutions was set to 25 °C. The viscosities were measured as a function of shear rate and no relevant changes were observed in the measured viscosities by linearly increasing the shear rate from 20 to 60 1/s. The tests were carried out in triplicate. The results were used for ranking the formulations relative to each other.

### 2.3. Electrospinning

A laboratory-scale electrospinning device—single-needle electrospinning (SNES) equipment—was used to screen the formulations. The selected formulations were scaled-up using high-speed electrospinning (HSES) technology. The scaled-up experiments were performed using the lab-scale and ultimately a pilot-scale HSES device.

To prepare a solution used for electrospinning, HPβCD, or HPβCD and ASO were added to the solvent and the mixture was stirred with a magnetic stirrer (100 rpm) at room temperature until complete dissolution. The HPβCD concentrations are indicated in *w*/*w*%, and solvent compositions are shown as a *v*/*v* ratio. The solution was fed with a syringe pump (SEP-10S Plus, Aitecs, Vilnius, Lithuania) and the feeding rate was adjusted for each solution to achieve adequate fiber formation.

SNES was equipped with a 0.5 mm inner-diameter nozzle, which was connected to a high-voltage supply. The electrical potential applied on the nozzle and the nozzle-collector distance were 25 kV and 30 cm, respectively. All of the experiments were carried out at room temperature.

Both of the HSES setups consisted of a round-shaped, stainless-steel spinneret connected to a high-speed motor [33]. The laboratory-scale HSES device was equipped with a spinneret (d = 34 mm) containing 8 orifices (d = 330 μm). The rotational speed of the spinneret was set to 10,000 rpm, and the voltage applied was 40 kV (Unitronik Ltd., Nagykanizsa, Hungary). A drying air flow (room temperature) and the electrostatic forces directed the fibers to the grounded metal collector covered with aluminum foil, which was at a fixed distance (35 cm) from the spinneret in a vertical arrangement. The pilot-scale HSES device consisted of a disk-shaped spinneret (d = 34 mm) containing 36 orifices (d = 330 μm) located in the side wall of the wheel. The rotational speed of the spinneret was fixed at 40,000 rpm. The applied voltage was 40 kV during the experiments (Quick 2000 Kft., Tiszavasvári, Hungary). The temperature of the drying air was adjusted in each experiment. The conical bottom of the drying chamber was grounded, and it acted as the counter electrode. Constant ventilation (120 m^3^/h) was applied to transport the fibers to the cyclone.

### 2.4. Scanning Electron Microscopy (SEM)

The morphology of the samples was studied by a JEOL 6380LA (JEOL, Tokyo, Japan) type scanning electron microscope. Samples were coated with gold using an ion sputter (JEOL 1200, JEOL, Tokyo, Japan) after being fixed with a conductive carbon adhesive tape. The accelerating voltage was set to 10 kV with a spot size of 50. Calculation of the average fiber diameter was performed based on a minimum of 70 measurement points using ImageJ (National Institutes of Health, Bethesda, MD, USA) software (version 1.53k).

### 2.5. FT-IR Spectroscopy

The spectral measurements were carried out by a Bruker Tensor 37 type Fourier transform infrared (FT-IR) spectrometer (Bruker Corporation, Billerica, MA, USA) equipped with a DTGS (deuterated triglycine sulfate) detector. The spectra were recorded in the range of 4000–400 cm^−1^ with a 4 cm^−1^ resolution. Before the infrared illumination, the reference and fibrous samples were mixed with potassium bromide (KBr) powder and cold-pressed into a suitable disk for transmission measurements.

### 2.6. Moisture Content Measurement

A Q5000 TGA instrument (TA Instruments, New Castle, USA) was used to determine the residual moisture content of the samples (*n* = 3, weighted between 4–6 mg) right after the electrospinning process and before HPLC analysis. Measurements were carried out under nitrogen atmosphere. A 10 °C/min heating speed was used to heat the samples from 25 to 105 °C, which were maintained at 105 °C for 10 min. A 50 mL/min nitrogen flush was applied during the measurement.

### 2.7. Methanol Content Measurements

Methanol content was determined with an Agilent 6890N gas chromatograph equipped with a CTC Combi PAL HS autosampler and coupled to an Agilent 5973 Inert mass spectrometer. Measurements were carried out on an HP-INNOWax (10 m × 0.25 mm × 0.25 µm) column with a headspace method. The temperature of the headspace needle was maintained at 140 °C and the incubation was also carried out at 140 °C. The incubation time was three minutes. Injections were carried out with a 25:1 split ratio with an injection volume of 250 µL. The oven program started at 35 °C and was maintained for 0.7 min. Afterward, the oven was heated to 200 °C with a constant rate of 35 °C/min and the final temperature was sustained for two minutes. The carrier gas was helium (purity: 99.999%) and the carrier flow was set to 1.7 mL/min. For detection, electron impact (EI) ionization was used with a 70 eV collision energy. The ion source and quadrupole temperatures were 230 °C and 150 °C, respectively.

### 2.8. Oligonucleotide Content

Oligonucleotide content in the samples was measured by reversed-phase high-performance liquid chromatography (RP-HPLC) (Agilent 1200 series LC System) after solving the fibrous formulations in phosphate-buffered saline. A gradient elution using mobile phase A (10% acetonitrile, 5 mM tributyl ammonium acetate, 1 μM ethylenediaminetetraacetic acid) and mobile phase B (80% acetonitrile, 5 mM tributyl ammonium acetate, 1 μM ethylenediaminetetraacetic acid) was performed at a flow rate of 1.44 mL/min and 40 °C for 32 min. The UV detection wavelength was set to 280 nm. A 5 μL sample volume (after dilution to approximately 1 mg/mL concentration) was injected into an Agilent Eclipse Plus C18 3.5 μm, 4.6 × 100 mm column.

### 2.9. Storage Stability Test

The prepared oligonucleotide-containing fibers were stored in a closed container at 4 °C and ~34% relative humidity. The relative humidity was controlled with a saturated MgCl_2_ solution. The oligonucleotide content of the samples was measured after electrospinning and 1, 3, 6, and 12 months of storage. The moisture content of the samples was measured right before HPLC analysis, and the calculated oligonucleotide content was corrected with the moisture content. The reference oligonucleotide formulation was maintained at −20 °C.

### 2.10. Downstream Processing of the Fibers to Tablets

The electrospun fibers were ground using a hammer mill (IKA MF10, IKA-WERKE GmbH & Co. KG, Staufen, Germany) with a 1 mm grid and 3000 rpm rotational speed. Direct compression tableting was carried out to prepare 600 mg round convex-shaped tablets from the ground oligonucleotide-loaded electrospun fibers with an eccentric CPR-6 tablet press, equipped with 14 mm concave punches (Dott. Bonapace, Limbiate, Italy). The tablets were prepared using the following composition: 31.5% MCC, 31.5% mannitol, 10.0% croscarmellose sodium, 1.0% silicon dioxide 1.0% magnesium stearate, and 25.0% fibrous powder. The compression force was about 12 kN.

### 2.11. Bulk and Tapped Density

Bulk and tapped density measurements of the grounded electrospun material were carried out using an ERWEKA SVM12 (Heusenstamm, Germany) type tester. The Hausner ratio and the Carr index (Carr, 1965; Hausner, 1967) were calculated and the flow property of the powders was determined.

### 2.12. Tablet Characterization

A Schleuniger 4M hardness tester (Thun, Switzerland) was used to measure the tablet breaking force of 10 tablets. The friability of 10 tablets was evaluated based on their average weight loss after 100 rounds in a Pharma Test PTF 20E (Hainburg, Germany) friability tester.

### 2.13. In Vitro Dissolution Test of the Tablets

A Pharmatest PTWS 600 dissolution tester (Pharma Test Apparatebau AG, Hainburg, Germany) was applied for investigating the dissolution of the tablets containing the oligonucleotide fibers. The Unites States Pharmacopoeia (USP) II (paddle) method was used during the experiments. The adjusted parameters were as follows: 100 rpm paddle rotational speed, 900 mL of dissolution media at pH 6.8, and 37 ± 0.5 °C. The ASO1 content was determined online through a flow cell system (equipped with 10 mm cuvettes) with an Agilent 8453 UV–Vis spectrophotometer (Agilent Technologies, Santa Clara, CA, USA). The software of the dissolution tester calculated in real-time the dissolution percentage based on a preliminary calibration at a wavelength of 258 nm. Two tablets were examined from each composition.

## 3. Results and Discussion

### 3.1. Effect of Methanol/Water Ratio and HPβCD Concentration on Electrospinning

**Figure 1:**
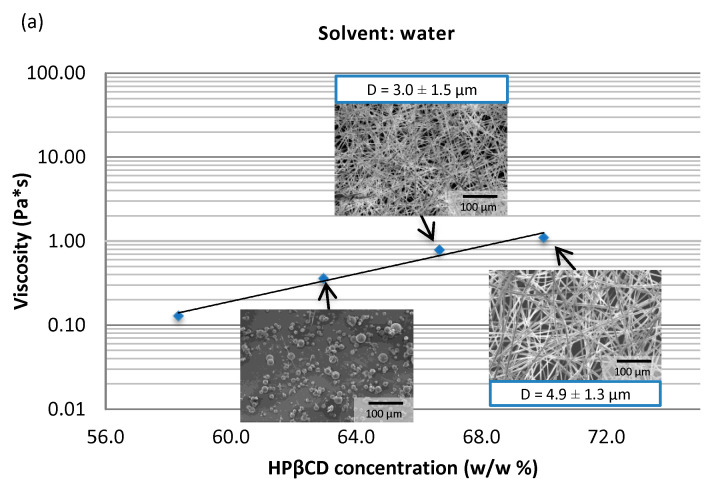

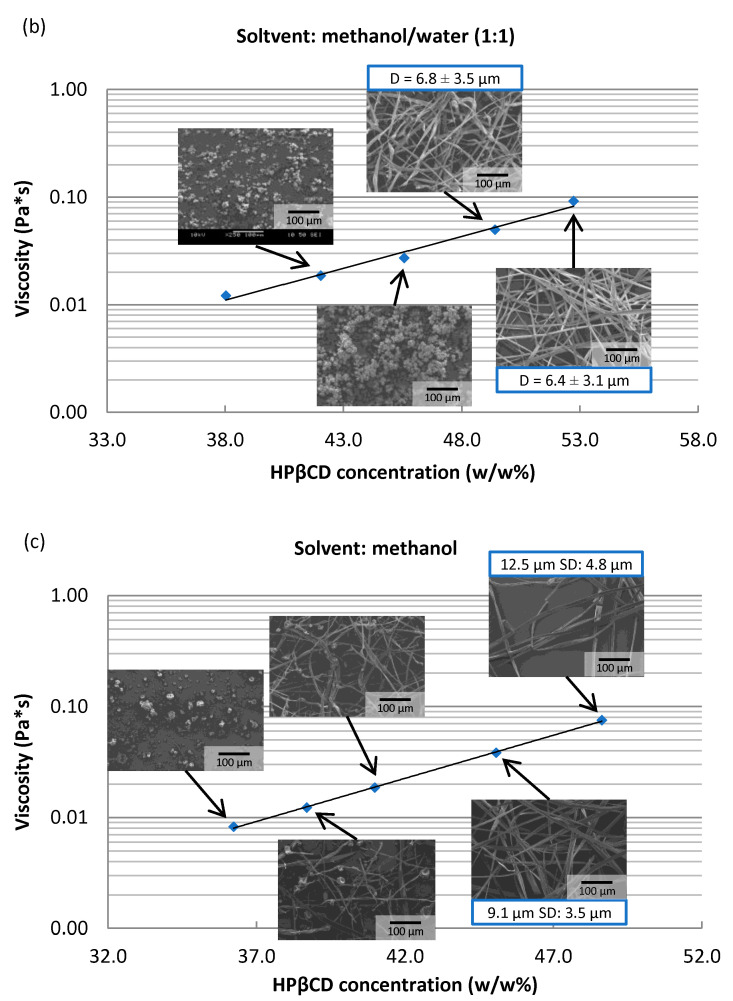
Viscosity of HPβCD solutions and the morphology of the obtained fibers prepared by SNES using (**a**) water, (**b**) methanol/water (1:1), (**c**) methanol as a solvent. D = average fiber diameter.

The electrospinning of HPβCD was tested in water, methanol, and methanol-containing aqueous solutions for the development of ASO-containing solid formulations. Different concentrations of HPβCD solutions were prepared in methanol (36.2, 38.7, 41.0, 45.1, 48.6%), in methanol/water (1:1) (38.0, 42.0, 45.6, 49.4, 52.7%), and in water (58.3, 63.0, 66.7, 70.0%) and electrospun using SNES. The viscosity of the solutions as a function of HPβCD concentration and the obtained fiber morphology are shown in Figure 1. As expected, the solutions at lower concentrations formed particles and by increasing the viscosity, fiber formation was achieved. As a result of electrospinning, depending on the composition and viscosity of the solution, bead-free fibers with different fiber diameters could be prepared. By changing the methanol ratio or the HPβCD concentration of the solutions, average fiber diameters between 3.0 and 12.5 µm were achieved. The fiber diameter increased with the methanol content of the solutions, which can be explained by the difference in solvent volatility. The lower boiling point of methanol helps faster evaporation and faster drying, resulting in thicker fibers.

The viscosity of the solutions at the suitable concentration range (where fiber formation took place) was between 0.02 and 0.08 Pa·s for methanol and 0.05 and 0.1 Pa·s for methanol/water (1:1). The viscosity values were much smaller compared to the aqueous solutions where the optimal viscosity range was between 0.5 and 1.0 Pa·s. Using methanol, electrospinning was possible at a lower viscosity and lower concentration range. This can be advantageous when high API loading is needed because the aqueous HPβCD solutions used for electrospinning already had a high viscosity and the handling and dosing of the solutions were difficult [34,38].

### 3.2. Scale-Up of HPβCD Electrospinning

Productivity is a critical factor when developing a drying technology in the pharmaceutical industry. However, during the electrospinning of aqueous HPβCD solutions using SNES technology, only 0.5 mL/h of feeding rate (0.5 g/h productivity) could be achieved. Using methanol as a solvent, the feeding rate of the electrospinning solution could be raised to 7 mL/h corresponding to 3.4 g/h of productivity (Table 1). The throughput of SNES technology is limited; thus, to increase productivity, HSES technology was utilized for scale-up.

Based on our previous studies with HSES, the concentration of HPβCD needs to be increased slightly to achieve adequate fiber formation when transferring a formulation from SNES to HSES [34]. Thus, solutions containing 70.0%, 52.7%, and 48.6% HPβCD in water, methanol/water (1:1), and methanol were used for scale-up, respectively. For the comparison of electrospinning using different solvents, a laboratory-scale HSES device was applied. The experiments were carried out to confirm the conclusion obtained using SNES as HSES has a different working principle.

During laboratory-scale HSES experiments, feeding rates up to 70 mL/h could be achieved using both methanol-containing HPβCD solutions and a 40 mL/h feeding rate was reached using the fully aqueous HPβCD solution. The electrospinning could be performed seamlessly using the developed placebo HPβCD formulations, and the productivity of the electrospinning was successfully increased (10–80 fold) by applying HSES technology. Furthermore, it can be possible to achieve even greater productivity in higher-dimensioned equipment.

**Figure 2:**
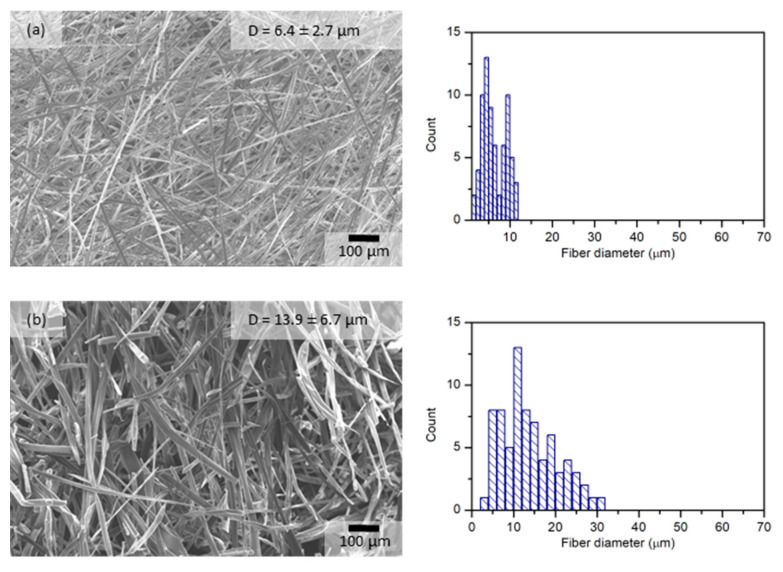

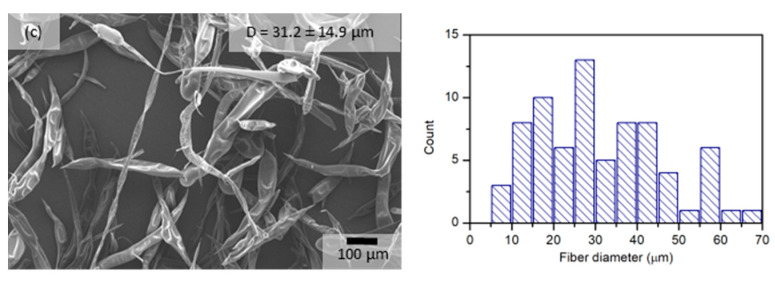
Comparison of fiber morphology and fiber diameter distribution of electrospun samples produced by lab-scale HSES equipment (40 kV, 10,000 rpm) using HPβCD solution containing (**a**) water, (**b**) methanol/water (1:1), and (**c**) methanol.

The effect of the solvent on fiber morphology after HSES was also evaluated (Figure 2) because it is an important factor during the downstream processing of the fibers. The fiber diameter of the methanol-containing formulations increased significantly compared to the water-based formulation. Furthermore, by increasing the methanol content of the solution from 50% to 100%, thus increasing the solvent’s volatility, the average fiber diameter increased from 13.9 µm to 31.2 µm. These results correlated with those obtained on the SNES device (Section 3.1).

### 3.3. High-Speed Electrospinning of ASO

The solid formulation of antisense oligonucleotides was attempted using HSES. To further increase productivity, a pilot-scale HSES device was utilized. ASO1 was used as a model to identify the best formulations and then applied to ASO2, which was available in limited quantities.

The placebo system optimization discussed in Section 3.2 was used as a basis for the solution preparations and electrospinning parameter settings. The ASO1 was not soluble in pure methanol but, using methanol/water (10:1), it was feasible to dissolve ASO1 besides the high concentration of HPβCD in a mass ratio of 1:10. This resulted in an API content of 9.1 *w*/*w*% in the solid formulations (Table A1). The addition of the ASO1 increased the viscosity of the solutions because the hydrogen bonds formed between oligonucleotides and HPβCD increased the entanglement of the molecules. Therefore, to be able to maintain appropriate fiber formation and solution handling, the HPβCD content was reduced in the API-containing solutions. The following formulations were prepared and electrospun using the pilot-scale HSES equipment:

- 62.5% HPβCD and 6.3% ASO1 in water.
- 47.6% HPβCD and 4.8% ASO1 in methanol/water (10:1).

The properties of the prepared solutions and the parameters used during the electrospinning process are summarized in Table 2. To achieve adequate drying, the temperature was set to 40 °C using water and to 30 °C using the methanol/water (10:1) solution. The collection of the fibers was accomplished continuously using a cyclone with good yields (70–80%), which can likely be improved by operating the equipment for a longer period.

The experiments showed that the productivity increased significantly (two orders of magnitude compared to SNES) using the pilot-scale HSES equipment. For the HPβCD–ASO1 formulations, ~330 g/h of productivity was successfully achieved.

**Figure 3:**
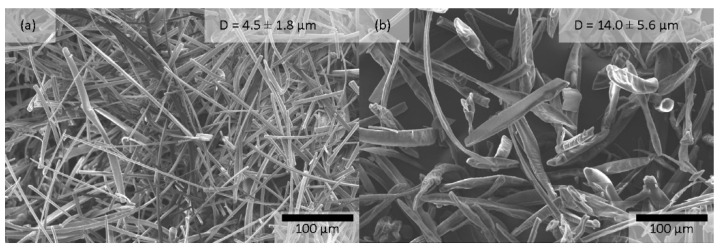
HPβCD–ASO1 formulations produced by pilot-scale HSES equipment from (**a**) water and (**b**) methanol/water (10:1). The electrospinning parameters are summarized in Table 2.

Similarly to the placebo experiments, adequate fibers were obtained without droplets, beads, or particle formation (Figure 3). The average fiber diameter was 4.5 µm for the 62.5% HPβCD + 6.3% ASO1–water formulation, and short fibers (average length of 92 µm) with an average fiber diameter of 14.0 µm were produced from the 47.6% HPβCD + 4.8% ASO1–methanol/water (10:1) formulation, whose fiber diameters were similar to those of the placebo formulations. The production of short fibers with a high average diameter can be an advantage during downstream processing. It can help to obtain an easy-handling material compared to nano-scale fibers (e.g., avoiding electrostatic charging of the fibers, better flowability). Furthermore, it could be possible to skip the grinding process during downstream processing.

The residual solvent content of the samples was measured with thermogravimetric analysis. The weight loss of the fibers electrospun from water, methanol/water (10:1), and the reference HPβCD powder was around 7.2 wt.%, 6.8 wt.%, and 6.5 wt.%, respectively (Table 3). The higher solvent content of the water-based formulation can be ascribed to the higher volatility of the solution. The methanol content of the formulation was also measured with gas chromatography. The 47.6% HPβCD + 4.8% ASO1–methanol/water (10:1) formulation contained 7400 ppm of methanol immediately after electrospinning. Owing to the high surface area of the fibers, with just an hour of room-temperature storage, the residual methanol content was below the ICH limit of 3000 ppm. The residual solvent of the samples was suitable, and thus, there was no need for secondary drying after electrospinning.

### 3.4. Long-Term Stability Study

**Figure 4:**
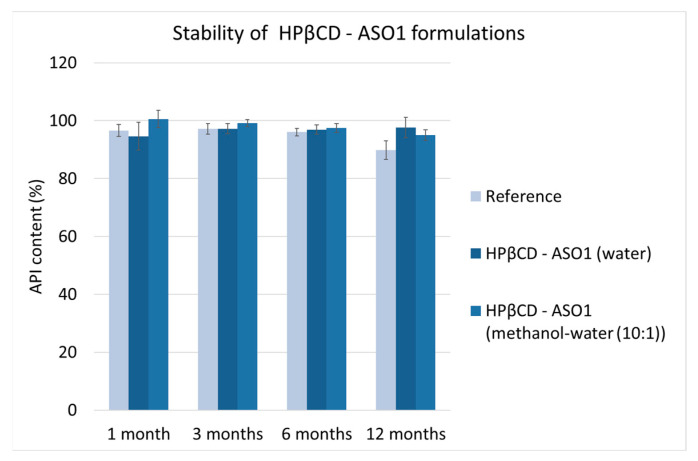
API content of ASO1-containing formulations during 12 months of storage.

**Figure A1:**
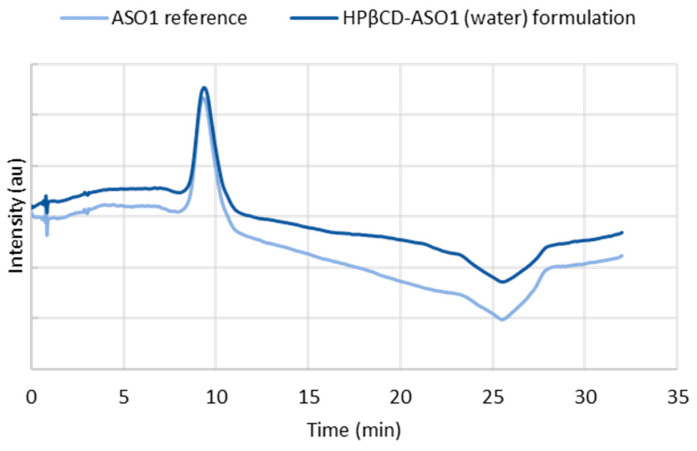
RP-HPLC chromatogram of ASO1 reference and HPβCD-ASO1 (water) formulation.

Maintaining the long-term stability of biopharmaceuticals is one of the main challenges in development. Therefore, new formulations need to stabilize biopharmaceutical agents to maintain their activity during storage. The stability of the electrospun HPβCD–ASO1 formulations were compared with pure, freeze-dried ASO1. The ASO1 content of the electrospun samples and the reference was measured after 1, 3, 6, and 12 months of storage at 4 °C and 34% relative humidity. For the reference material, the chromatogram showed a single, clear peak. The chromatogram of the electrospun formulations containing ASO1 was very similar, and no extra peaks or peak deformations were observable (Figure A1). The API content of the ASO1 formulations, which indirectly provides information on stability, is shown in Figure 4.

The 1-year stability measurement showed that the electrospun formulations preserved their stability and there was no notable change in API content. On the contrary, the ASO1 content of the reference formulation decreased to 89%. The results suggest that the chosen cyclodextrin is a suitable matrix for the formulation of oligonucleotides; thus, stable formulations of ASO1 could be prepared using HPβCD by high-speed electrospinning.

### 3.5. FT-IR Analysis of ASO-Containing Fibers

**Figure 5:**
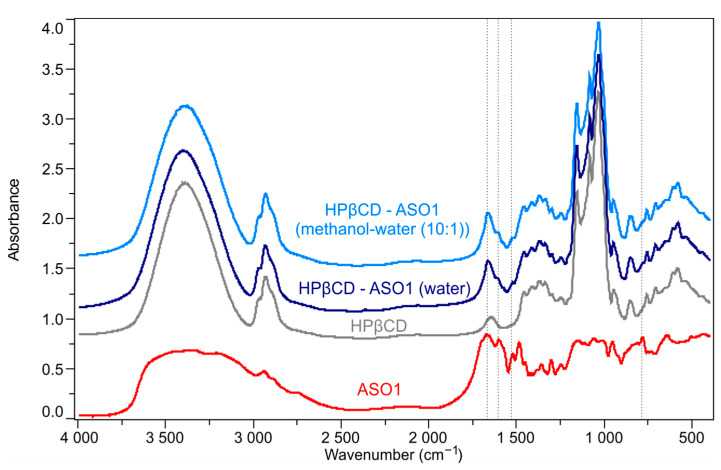
FR-IR spectra of the reference (ASO1 and HPβCD) and fibrous materials (HPβCD–ASO1) formulations.

The FT-IR spectra were recorded to identify characteristic absorbances of ASO1 in the electrospun HPβCD–ASO1 formulations. The reference spectra of the two starting materials differ from each other. It is worth mentioning that HPβCD has a very strong absorbance in the ranges of 3600–3000 (O-H stretching) and 1200–1000 cm^−1^ (C-O and C-O-C stretching). These vibrations masked the weaker signals of ASO1. However, FT-IR showed that several vibrational bands of ASO1 modified the HPβCD absorbances in the formulations. Figure 5 illustrates that the bands at 1664, 1600, 1522, and 781 cm^−1^ appeared in the case of HPβCD–ASO1 formulations, where only slight overlapping occurred, confirming API content.

### 3.6. Processing of ASO-Containing Fibers to Tablets

**Figure 6:**
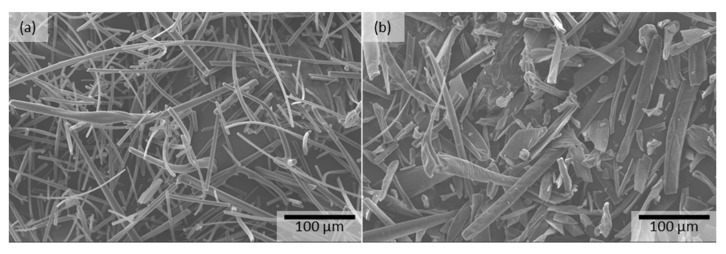
Ground fibers using a hammer mill: (**a**) HPβCD-ASO1–water; (**b**) HPβCD-ASO1–methanol/water (10:1) formulation.

Downstream processability of the electrospun drug-loaded fibers (e.g., milling, powder properties, tableting) is critical in the development of solid oral pharmaceutical products. Although fragmentation of the fibers occurred during electrospinning of 47.6% HPβCD + 4.8% ASO1–methanol/water (10:1), the produced fibers formed larger agglomerates; thus, the product was not suitable for further processing. Therefore, the milling of the HPβCD-ASO1 fibers was carried out right after electrospinning by means of a hammer mill. The grindability of the oligonucleotide-containing fibers was excellent, and the milling of the fibrous sample resulted in a powder. Owing to the non-sticky nature of the fibers, the yield of the milling was between 96 and 98%. The SEM images show that the fibers were broken into smaller fragments, but the formulations maintained their fibrous structure (Figure 6).

Among the powder characteristics, flow properties are the most important ones considering the downstream processability of the fibers. Thus, the bulk and tapped densities were measured and the Hausner ratio and Carr index were calculated for each formulation (Table 4). The ground fibers showed poor flow characteristics and a fluffy nature. Nevertheless, it was possible to improve powder properties by mixing the ground fibers with excipients (Section 2.9).

The HPβCD-ASO1 ground fibers electrospun from methanol/water (10:1) had a higher fiber diameter (14.0 µm) compared to the fibers electrospun from water (4.5 µm), resulting in a higher bulk and tapped density and better flowability. It was also possible to perform automatic tableting using the powder mixture containing HPβCD-ASO1–methanol/water (10:1) formulation.

HPβCD-ASO1-containing tablets were prepared successfully from electrospun fibers after grinding and mixing. Approximately 30 tablets were prepared from each formulation and the tablet properties were characterized (Table 5). The tablets had good hardness and friability (weight loss was lower than 1%).

**Figure 7:**
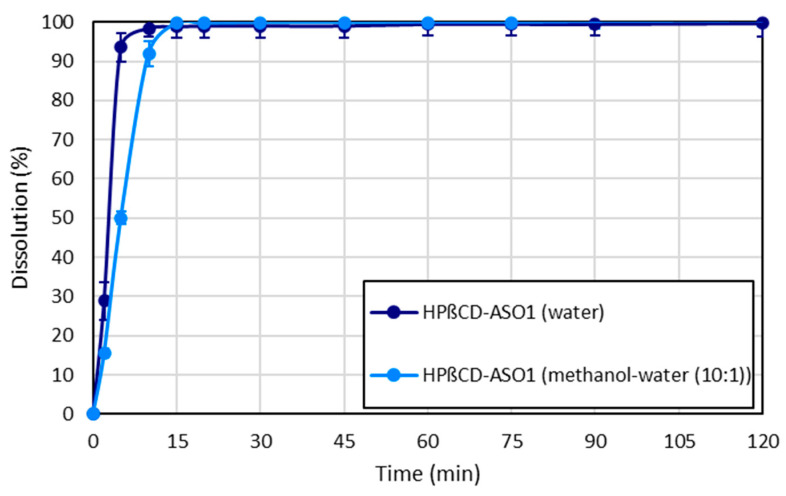
The dissolution profile of the tablets containing oligonucleotide fibers (900 mL of pH 6.8 dissolution media, 37 ± 0.5 °C, USPII method, 100 rpm, 258 nm, 13.5 mg of ASO1 content, *n* = 2).

It is important to note that antisense oligonucleotides are sensitive biomolecules that are prone to degradation in the GI tract by low pH and enzymatic activity. To use the developed tablet formulation, an acid-resistant coating should be used to protect the API until it reaches the colon. For this purpose, cellulose acetate phthalate, HPMC acetate succinate, or Eudragit L/S is conventionally used in the pharmaceutical industry as a coating agent [39], but the development of the coating technology was not the aim of this study. However, for the above-mentioned reasons, the dissolution of the tablets was investigated in the dissolution media of pH 6.8 to simulate the relevant media corresponding to these formulations. Immediate release of the ASO1 from both tablets was successfully achieved according to the dissolution tests (Figure 7). The disintegration time of the tablets containing HPβCD-ASO1 (water) and HPβCD-ASO1 (methanol-water (10:1)) fibers was 3 and 7 min, respectively. The disintegration time and the dissolution percentage were well correlated with the fiber diameter as the fibers with smaller diameters (HPβCD-ASO1 (water)) disintegrated earlier and reached a higher dissolution percentage in the first 10 min.

### 3.7. Development of High-API-Loading Electrospun Formulation

Currently, the oral dose of an antisense oligonucleotide is high because of its low bioavailability. To meet the demands of a high-dose formulation, the oligonucleotide content of the fibers was increased and solutions were prepared with a 50% API ratio for electrospinning (Table A2). After obtaining a fiber-forming and easy-to-process system with ASO1, the formulations were transferred and modified to obtain an adequate formulation with ASO2 with the aim to develop high-loading formulations.

Regardless of the chemical similarity of the APIs, ASO2 precipitated in the presence of HPβCD in methanol/water (10:1). To increase the solubility, the methanol ratio of the solvent was decreased. Using methanol/water (3:1), it was possible to make a clear solution containing ASO2 and HPβCD. Besides increasing the API ratio of the formulations to 50%, the total solid content of the solutions was decreased (20%, 30%, and 40%) because the viscosity of the samples was too high.

**Figure 8:**
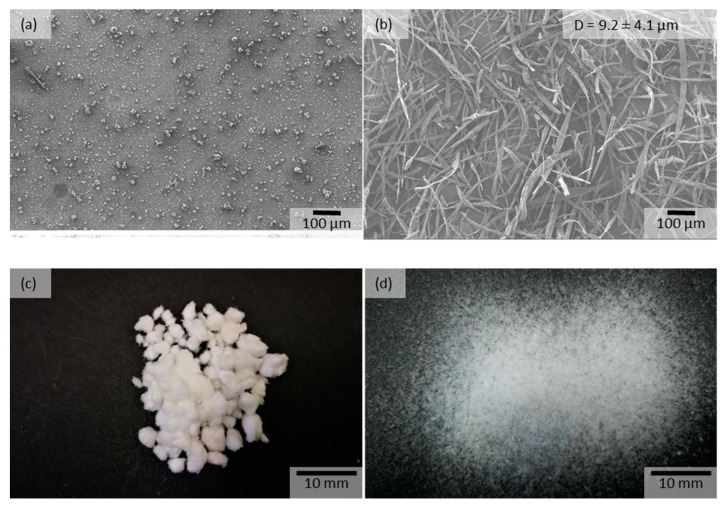
SEM image of the particles obtained from the electrospinning of 40% (HPβCD-50%ASO2) methanol/water (3:1) solution using the (**a**) laboratory-scale HSES equipment with 10,000 rpm, 40 kV, and 20 mL/h, and the (**b**) pilot-scale HSES equipment with 40,000 rpm, 40 kV, and 100 mL/h. Photo of the fibers (**c**) before and (**d**) after sieving (only tapping).

The electrospinning of the 40% (HPβCD–50% ASO2)–methanol/water (3:1) solution using the laboratory-scale HSES equipment resulted in particles; however, by using the pilot-scale device, it was possible to produce fibers from the same formulation (Figure 8a,b). The rotational speed of the spinneret was higher using the pilot-scale device, which resulted in higher elongation forces and faster evaporation. If the evaporation is slower, there is a chance for droplet formation from the jet due to the driving force of the surface tension. The electrospinning had good productivity (105 g/h) and the fibers could be collected by a cyclone. The average fiber diameter was around 9.2 µm for the HPβCD–50% ASO2 formulation. The produced short fibers (with an average length of 228 µm) had excellent grindability and were easily separated by tapping a sieve (no pushing, Figure 8c,d). The short fibers produced by HSES can be used directly for tableting without the need for milling or grinding. This can be advantageous in general, but especially during the development of a continuous manufacturing line.

The development of the 50% ASO2 formulation made it possible to prepare tablets with higher API content. A 600 mg tablet containing 25% fibers would have a dose of 125 mg of API, which is a ninefold increase compared to the initial formulation.

## 4. Conclusions

The presented work proves that HSES is a suitable technology for the scaled-up production of HPβCD fibers for the delivery of an antisense oligonucleotide. The electrospinning of HPβCD was compared from water, methanol/water (1:1), and methanol using SNES based on viscosity and morphology. Furthermore, to meet the productivity requirements of the pharmaceutical industry, the scale-up of electrospinning was carried out using HSES technology. The productivity of the process could be increased up to 330 g/h using pilot-scale HSES equipment connected to a cyclone.

The average fiber diameter was 4.5 µm for the 62.5% HPβCD + 6.3% ASO1–water formulation. However, the electrospinning of 47.6% HPβCD + 4.8% ASO1–methanol/water (10:1) solution resulted in the production of short fibers with high average fiber diameter (14.0 µm), due to the high volatility of the solvent. Grindability and flowability of the fibers are important factors of downstream processability and were, therefore, investigated. The API-containing formulations had excellent grindability and the obtained powder maintained the fibrous structure. However, the powder had poor flowability, which could be improved by mixing the ground fibrous powder with excipients. The increased fiber diameter due to the use of methanol enhanced the processability of the fibers and automatic tableting could be performed using the HPβCD-ASO1 (methanol-water (10:1)) formulation. On the other hand, the dissolution of the tablets prepared from fibers with larger fiber diameters resulted in increased dissolution and disintegration time. The fibrous HPβCD–ASO1 formulations were stored at 4 °C and 34% RH for a year to assess the stability of the oligonucleotide. ASO1 showed no sign of degradation over the 1 year of storage, which also shows the suitability of the HPβCD matrix for the formulation of biopharmaceuticals.

The developed formulation compositions for ASO1 were transferred and modified to obtain an adequate formulation using another oligonucleotide, ASO2. The results could draw attention to the challenges of formulation transfer. Furthermore, to meet the demand for high-dose formulations, HPβCD–ASO2 fibers were successfully prepared with an API loading of 50%. The electrospinning resulted in the production of short fibers, which could be processed without the need for grinding. This can be advantageous when designing a manufacturing line for biopharmaceutical production.

In conclusion of this work, the gentle drying by HSES and the excellent processability of the formulation enabled the preparation of a tablet dosage form for the oral administration of model oligonucleotide drugs. Although oral tablet formulations were developed in this work, cyclodextrins are injectable excipients, so electrospun formulations of cyclodextrins can be used in other dosage forms (e.g., powder for reconstitution).

## Figures and Tables

**Table 1:** Comparison of the productivity achieved using SNES and HSES equipment with HPβCD solutions. Productivity was calculated from the feeding rate, solution density, and HPβCD content. The density of the solutions containing 70.0% HPβCD in water, 52.7% HPβCD in methanol/water (1:1), and 48.6% HPβCD in methanol was 1.36 g/mL, 1.14 g/mL, and 1.01 g/mL respectively.

Productivity (g/h)–Feeding Rate (mL/h)			
Formulation/ Preparation Method	70.0% HPβCD Water	52.7% HPβCD Methanol/Water (1:1)	48.6% HPβCD Methanol
SNES	0.5 g/h–0.5 mL/h	3.0 g/h–5 mL/h	3.4 g/h–7 mL/h
Laboratory-scale HSES	38.2 g/h–40 mL/h	41.9 g/h–70 mL/h	34.2 g/h–70 mL/h

**Table 2:** Electrospinning parameters during pilot-scale HSES. The rotational speed of the nozzle was set to 40,000 rpm and 40 kV was applied to it in each experiment. Ventilation (120 m^3^/h) was used to collect the fibers in the cyclone.

Solution	Viscosity (Pa·s)	Density (g/mL)	Feeding Rate (mL/h)	Productivity (g/h)	Electrospinning Temp. (°C)
62.5% HPβCD + 6.3% ASO1 water	1.13	1.23	400	338	40
47.6% HPβCD + 4.8% ASO1 methanol/water (10:1)	1.70	1.05	600	331	30

**Table 3:** Residual solvent content of the electrospun formulations.

Formulation	Total Solvent Content (wt.%)	Methanol Content (ppm) Just after the Production	Methanol Content after 1 h at RT (ppm)
62.5% HPβCD + 6.3% ASO1 water	7.2	-	-
47.6% HPβCD + 4.8% ASO1 methanol/water (10:1)	6.8—split in Water 6.1 wt.% and Methanol 0.7 wt.%	7400	<3000

**Table 4:** Comparison of flowability: HPβCD containing formulations with different fiber morphology and their mixture with tableting excipients.

Formulation	Powder Composition	Bulk Density (g/L)	Tapped Density (g/L)	Hausner Ratio	Carr Index (%)
HPβCD-ASO1 water	fibers	0.077	0.130	1.69	41
	25% fibers mixed with excipients	0.341	0.531	1.56	36
HPβCD-ASO1 methanol/water (10:1)	fibers	0.167	0.308	1.84	46
	25% fibers mixed with excipients	0.422	0.623	1.48	32

**Table 5:** Properties of ASO1-containing tablets calculated as an average of 10 tablets.

Formulation	The Fiber Content of Powder (%)	Compression Force (kg)	Individual Weight (mg)	Tablet Breaking Force (N)	Weight Loss (%)
HPβCD-ASO1–water	25	1157 ± 29	592.6 ± 9.9	139 ± 10	0.63
HPβCD-ASO1–methanol/water (10:1)	25	1214 ± 294	602.3 ± 34.8	145 ± 11	0.67

## Data Availability

Not applicable.

## Appendix A

**Table A1:** Composition of solutions used for electrospinning. The methanol density (0.792 g/cm^3^) was used for the calculations.

Solution Composition	HPβCD (g)	ASO (g)	API Ratio of the Total Solid Content (*w*/*w*%)	Solvent (g)	Water (mL)	Methanol (mL)
62.5% HPβCD 6.3% ASO1 water	80	8	9.1	40	40.0	-
47.6% HPβCD 4.8% ASO1 methanol/water (10:1)	80	8	9.1	80	8.97	89.7

**Table A2:** Solution preparation of ASO2 and HPβCD in methanol–water. The electrospinnability of the so-lutions was tested using laboratory-scale HSES equipment.

Total Solid Content of the Solution (*w*/*w*%)	API Ratio of Total Solid Content (*w*/*w*%)	Solvent (*v*/*v*)	Clear Solution?	Electrospinnability
16	100	methanol	yes	-
20	10	methanol/water (10:1)	no	-
20	50	methanol/water (10:1)	no	-
30	50	methanol/water (10:1)	no	-
20	50	methanol/water (3:1)	yes	no
30	50	methanol/water (3:1)	yes	no
40	50	methanol/water (3:1)	yes	particles < 10 µm
